# Prediction of complications in diabetes mellitus using machine learning models with transplanted topic model features

**DOI:** 10.1007/s13534-023-00322-7

**Published:** 2023-10-06

**Authors:** Benedict Choonghyun Han, Jimin Kim, Jinwook Choi

**Affiliations:** 1 Interdisciplinary Program in Bioengineering, Seoul National University, 1 Gwanak-ro Gwanak-gu, Seoul, 08826 Republic of Korea; 2 English Language and Literature, Seoul National University, 1 Gwanak-ro Gwanak-gu, Seoul, 08826 Republic of Korea; 3 Department of Biomedical Engineering, College of Medicine, Seoul National University, 101 Daehak-ro Jongno-gu, Seoul, 03080 Republic of Korea; 4 Institute of Medical and Biological Engineering, Medical Research Center, Seoul National University, 103 Daehak-ro Jongno-gu, Seoul, 03080 Republic of Korea

**Keywords:** Diabetes Mellitus, Latent Dirichlet allocation, Machine learning, Topic modeling

## Abstract

**Purpose**: This study aims to predict the progression of Diabetes Mellitus (DM) from the clinical notes through machine learning based on latent Dirichlet allocation (LDA) topic modeling. Particularly, 174,427 clinical notes of DM patients were collected from the electronic medical record (EMR) system of the Seoul National University Hospital outpatient clinic. **Method**: We developed a model to predict the development of DM complications. Topics developed by the topic model were exploited as the key feature of our machine-learning model. The proposed model generalized a correlation between topic structures and complications. **Results**: The model provided acceptable predictive performance for all four types of complications (diabetic retinopathy, diabetic nephropathy, nonalcoholic fatty liver disease, and cerebrovascular accident). Upon employing extreme gradient boosting (XGBoost), we obtained the F1 scores of the predictions for each complication type as 0.844, 0.921, 0.831, and 0.762. **Conclusion**: This study shows that a machine learning project based on topic modeling can effectively predict the progress of a disease. Furthermore, a unique way of topic model transplanting, which matches the dimension of the topic structures of the two data sets, is presented.

**Supplementary Information:**

The online version contains supplementary material available at 10.1007/s13534-023-00322-7.

## Introduction

Diabetes Mellitus (DM) is a lifetime disease that requires recurrent hospital visits. According to the World Health Organization, more than 400 million patients worldwide suffer from DM [1]. Over time, high or low blood sugar levels can interfere with regular body functions, including those of the kidneys, eyes, feet, and other organs [1]. Appropriate management of diabetes is critical for ascertaining quality of life, especially in middle-aged patients. Thus, in patients with DM, predicting its progression at an early stage is important. Therefore, we aim to predict the progression of diabetic complications using a semi-supervised classification model based on latent Dirichlet allocation (LDA).

We trained the model on the topic structure of the clinical notes of patients with DM collected from the electronic medical record (EMR) system of the Seoul National University Hospital (SNUH) outpatient clinic. Furthermore, we input their complication status data into the model, which yielded a generalized correlation between topic structure and complication status. Subsequently, by entering the transplanted topic feature of the held-out test data into the model, we attempted to compute the probability of future complications. The model performed well in predicting complications, proving the effectiveness of the current approach.

Recent studies have focused on predicting DM complications. Thomas et al. collected the records of previous diagnoses, medical history, and demographic information (including age, sex, and laboratory test results) of patients with type 2 DM, using which they inferred the onset of diabetes complications [2]. Ljubic et al. collected the diagnostic records of patients with type 2 DM for each hospitalization [3]. They applied a one-way recurrent neural network and a bidirectional recurrent neural network (RNN)-gated recurrent unit to predict ten complications: angina pectoris, atherosclerosis, ischemic heart disease, depressive disorder, diabetic nephropathy (DMN), diabetic neuropathy, diabetic retinopathy (DMR), hearing loss, myocardial infarction, and peripheral vascular diseases [3].

The researchers in the aforementioned studies collected data directly from particular fields in the database to fill in predefined feature sets. However, the clinical notes may contain hidden clues for the inference of future disease progress. Therefore, an inductive data-driven approach was proposed. In particular, we employed a topic modeling method to detect the information contained in the records collected.

Topic modeling, or LDA, is a dimensionality reduction method developed by Blei et al. [4]. It is a statistical method that analyzes words in original documents to discover the themes running through them and the interconnection of these themes [5]. Several relevant studies have been conducted since Papadimitriou, Raghavan, Tamaki, and Vempala first proposed latent semantic indexing (LSI) in 1998 [6]. Hofmann also proposed a method that replaced term frequencies (TF) with the probability of word occurrence [7]. As an extension of Hofmann’s work, a generative probabilistic topic model, also known as LDA, was proposed by Blei et al. Their topic modeling was based on a variational expectation-maximization (EM) algorithm [4]. As an alternative model to that of Blei et al., Griffiths et al. proposed an approach for LDA that utilizes the Gibbs sampling algorithm [8].

The LDA has been applied to various tasks in clinical text processing. Perotte et al. proposed a risk prediction model for chronic kidney disease (CKD) progression by incorporating topic models of clinical documents and accumulated laboratory test results to obtain more accurate prediction results [9]. They showed that topic models could serve as effective independent variables for disease models predicting CKD progression. Sarioglu et al. performed support vector machine (SVM) classification on topic models estimated from radiology notes to identify patients diagnosed with orbital fractures [10]. For classification, they extracted features from topic modeling. Restificar applied topic modeling to a task comprising the eligibility criteria for clinical trials [11]. Halpern et al. performed text processing using topic models estimated from triages recorded by nurses in the emergency room (ER) to identify patients who had a high risk of infection, which might cause fatal diseases, such as sepsis [12]. They applied many types of dimensionality reduction methods to determine the most effective method.

In this study, we presume the topic to represent hidden clues. A topic is extracted from a document using a reductive modeling procedure, which can be expressed as a cluster incorporating semantically related words [4]. Topics are important for predicting or classifying data. These studies show that topics can act as independent variables in prediction models [9–12]. When a researcher expects that a particular property of a data entity may be an influential feature affecting a phenomenon of interest, statistical analysis should be performed to define a numerical independent variable representing this property. This procedure requires rigorous data analysis. Topic modeling is further expected to simplify the data analysis procedure.

The remainder of this paper is organized as follows: Sect. 2 describes the data and methodologies used in this project. In Sect. 3 the results are analyzed and discussed in more detail in Sect. 4. Finally, concluding remarks are provided in Sect. 5.

## Material and method

**Fig. 1:**
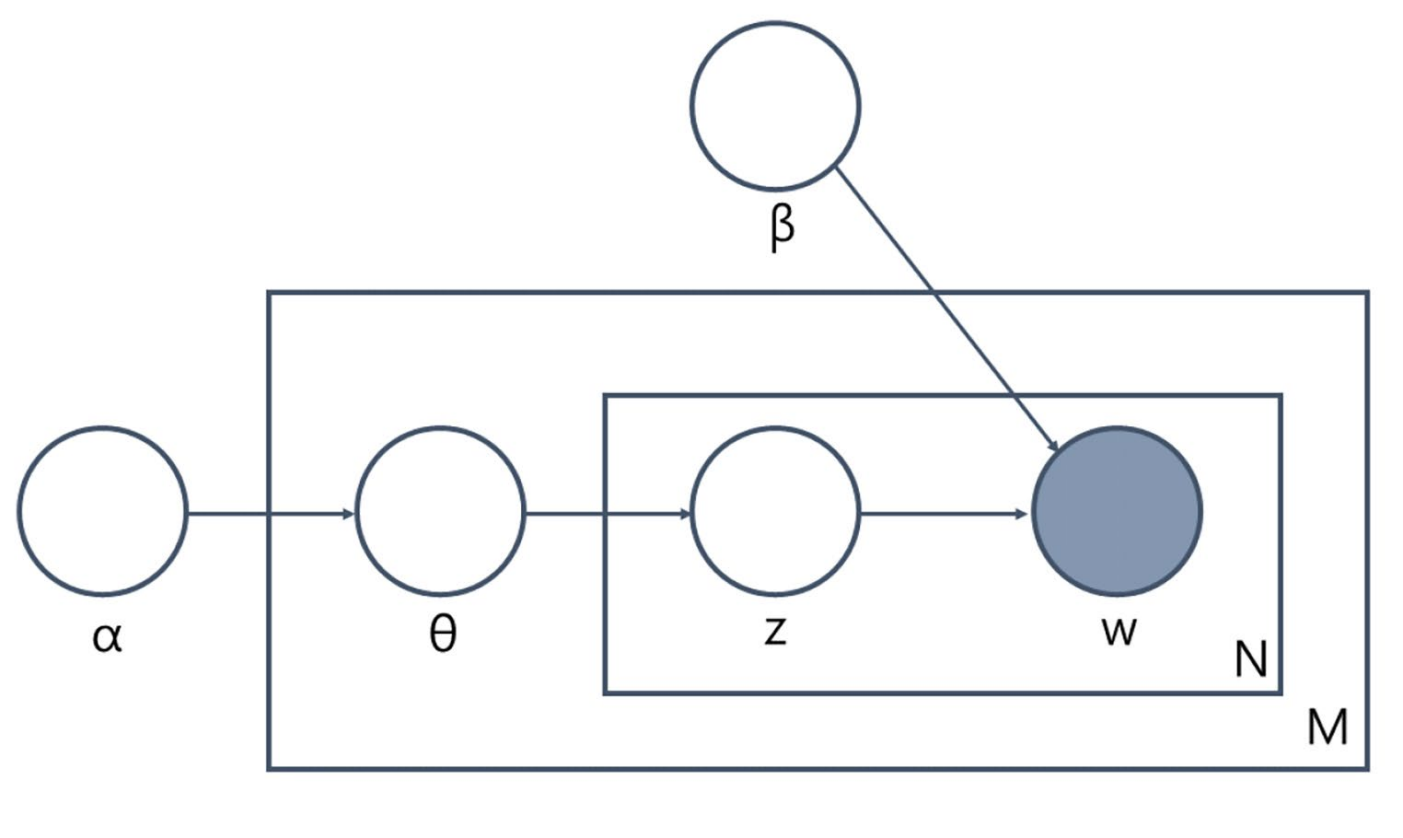
Basic concept of LDA [4] *α*: Initial parameter to Dirichlet distribution; *β*: parameter signifying the relations between words and topics; *θ*: parameter containing the relations between documents and topics; M: number of documents in a dataset; N: number of words in a document; w: words appearing in a document; Z:topic allocations to words in a document

As aforementioned, LDA was the basic approach to processing the clinical notes of our data. To provide a brief background on LDA, it is assumed that every word (w) in the actual document (d) is produced under the influences of *θ* and *β* (see Fig. 1). α is an initial parameter to Dirichlet distribution. *θ* expresses the document-topic relation, and *β* reflects the word-topic relation. Z is an example of the effects of the influences of *θ* and *β*. This includes pairs of words and topic numbers, indicating the assignment of a word to a particular topic in the document.

The gray circle in Fig. 1 represents the actual occurrence of a word, while the transparent circles represent hidden or abstract objects. Topic modeling is a posterior procedure estimating the approximate parameters of *θ* and *β* from a data set. Thus, the dataset can be translated into a matrix (M × K), where M represents the number of documents, and K is the topic count. The matrix (M × K) is called the topic structure.

The basic approach of this study aimed to predict the onset of DM-related complications using the clinical notes of patients through a semi-supervised classification model. LDA or topic modeling was employed to reduce the dimensions of the input data. Topic modeling is advantageous because it reduces the dimension of the TF matrix filled with 0s, which provides a memory space benefit. The data of the patients were grouped according to four types of well-known DM complications. In each group, an analogous number of positive cases (i.e., patients with DM who developed complications) and negative cases (i.e., patients with DM who did not develop complications) were included. This enabled the subsequent computation of the correlation between topic structure and complications. In each group, 90% of the data were used to train the classification model, and the remaining 10% were used as test data.

After the training data were text processed and indexed, they were organized into a document-term matrix (M × V). Through topic modeling, this matrix was converted into a document-topic matrix (M × K) that demonstrated the estimated topic structure. The topic structure and complication information of the training data were entered into the classification model, which then computed the correlation between them.

**Fig. 2:**
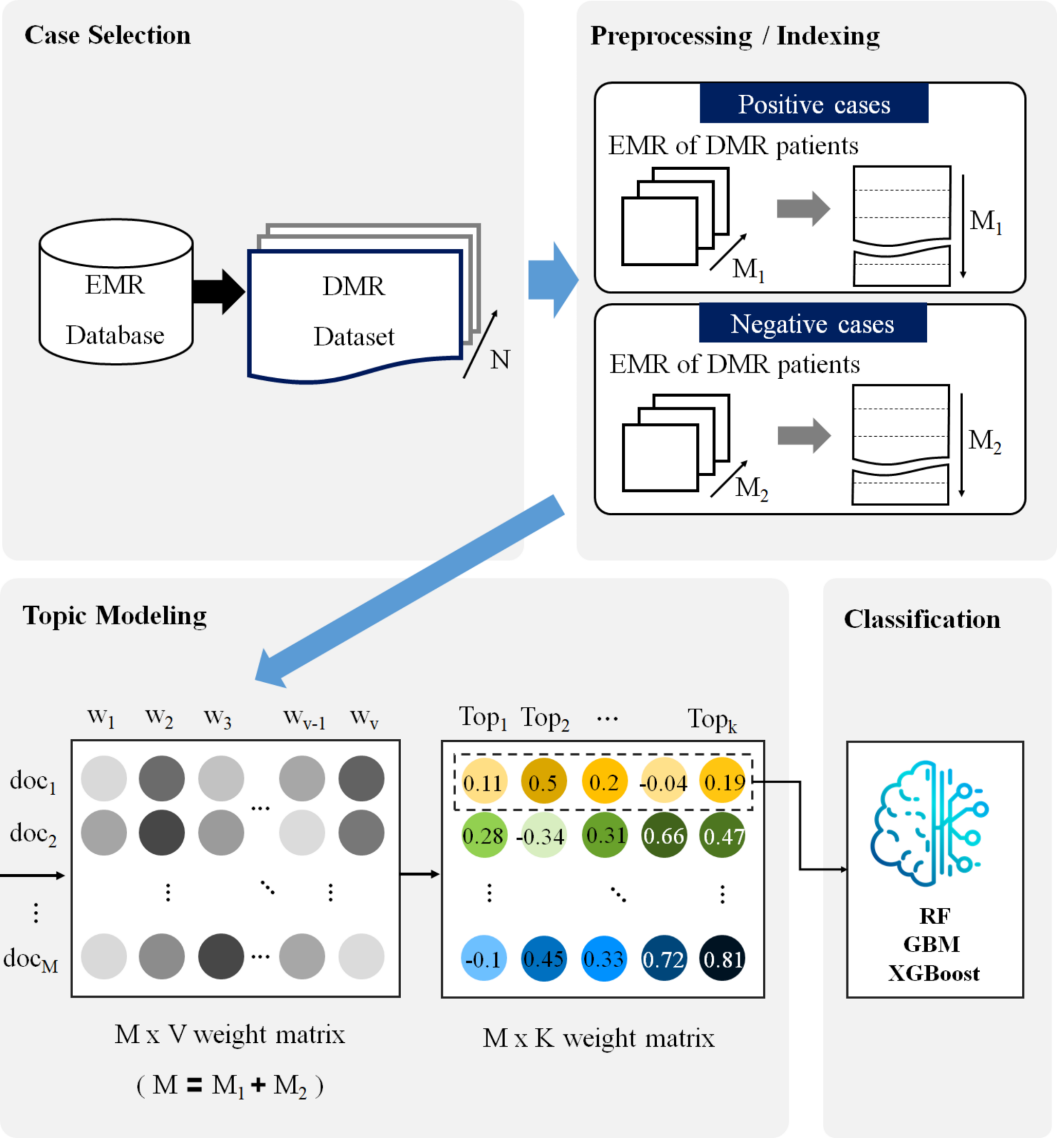
Overall Workflow Preprocessing: correcting typos, part of speech (POS) tagging, composing stop words list, and replacing drug product names with ingredient names; Indexing: filling a matrix (M × V) with term frequency(TF) values; Topic Modeling: filling a matrix (M × K) with the document-topic weight values; Classification: predicting the label variable utilizing the machine learning model

Subsequently, based on the topic structure of the training dataset, the weighted topic structure of the test data was computed, referred to as the transplanting process. Therefore, we matched the structures of the training and test data. The weighted topic structure of the test data was inputted into the designed classification model. The classification model automatically computed the probability of complications in the test data based on the trained correlation between the topic structure and the onset of complications. Figure 2 illustrates the overall workflow of this study explained. Data acquisition from the SNUH EMR systems precedes pre-processing/indexing. The preprocessing included tokenization and part of speech (POS) tagging. Tokenization splits sentences into tokens, POS tagging identifies POS properties, and POS tags are attached to each token. Indexing counts the TF of each word in a document and composes an M × V matrix. Topic modeling accepts an M × V matrix as the input variable to produce an M × K matrix. Examples of the M × V and M × K matrices are included in the supplement.

### Data set

The clinical notes collected for this study were text documents written by clinicians in the outpatient clinics while treating patients. These generally contain the medical history of the patient, chief complaint, physical examination results, test results, impression, and a plan describing subsequent examinations and medications. We obtained the clinical notes of 9,430 patients with DM from the EMR system of the SNUH outpatient clinic, from 2013 to 2015. Furthermore, we collected diagnostic data for these patients from their outpatient clinic visits between 2013 and 2020. Data collection was approved by the Institutional Review Board (IRB) of Seoul National University Hospital (IRB NO: C-1612-085-815). Thereafter, we divided the data into four groups according to the type of DM complication: diabetic retinopathy (DMR), diabetic nephropathy (DMN), nonalcoholic fatty liver disease (NAFLD), and cerebrovascular accident (CVA). To analyze the correlation between the topic structure of the data and complications, negative cases were included in each group of data. The numbers of positive cases (i.e., DM patients who developed complications) and negative cases (i.e., DM patients who did not develop complications) in each group were balanced. For topic modeling, clinical notes of three years for each patient were merged into a single document. The average number of visits for positive cases in each group is described in each subsection.

#### DMR data Set

The DMR group comprised 1,747 patients diagnosed with DMR ( positive cases) and 1,653 patients with DM who did not develop DMR ( negative cases). The ICD-10 codes used to identify the dataset were E14.3 (diabetic retinopathy), H36.0 (nonproliferative diabetic retinopathy), and E11.3 (type 2 diabetes mellitus with non-proliferative retinopathy). On average, the patients visited the outpatient clinic 13.9 times between the first diagnosis date of DM and that of DMR.

#### DMN data Set

Using ICD-10 codes E14.2 (unspecified diabetes mellitus with renal complications) and E11.2 (diabetes mellitus with kidney complications), 970 patients with DM diagnosed with DMN were included in the DMN group. In total, 997 negative cases were included in this group. The average number of visits to the outpatient clinic by DMN-positive patients in this group was 20.8 times between the first diagnosis of DM and that of DMN.

#### NAFLD data Set

In the NAFLD group, 444 patients with DM and NAFLD were selected as positive cases. In total, 411 negative cases were included. The ICD-10 codes used to obtain these data were K75.8 (nonalcoholic steatohepatitis) and K76.0 (fatty liver). NAFLD-positive patients in this group visited the outpatient clinic 13.2 times on average, between the first diagnosis of DM and that of NAFLD.

#### CVA data Set

In the CVA group, 401 patients also diagnosed with CVA were selected as positive cases. There were 407 negative cases in this group. The ICD-10 codes I63.9 (cerebral infarction, unspecified) and I63.8 (other cerebral infarctions) were used to obtain this dataset. The CVA-positive patients in this group visited the outpatient clinic 15.2 times on average, between the first diagnosis of DM and that of CVA.

Table 1 summarizes the properties of each dataset. As shown in this table, the proportions of positive and negative cases in each group were balanced.

**Table 1:** Properties of Datasets

Dataset	M^*a*^	V^*b*^	BC^*c*^	pos^*d*^	neg^*e*^	Av^*f*^
DMR	3,400	14,316	99.50%	51.40%	48.60%	13.9(11.14)
DMN	1,967	12,073	99.30%	50.70%	49.30%	20.8(12.19)
NAFLD	855	8,225	99.30%	51.90%	48.10%	13.2(11.08)
CVA	808	8,453	99.30%	49.60%	50.40%	15.2(15.01)

^*a*^ number of merged documents, ^*b*^ vocabulary size, ^*c*^ percentage of blank cells in M by V matrix, ^*d*^Percentage of positive cases, ^*e*^ Percentage of negative cases, ^*f*^ average visit count (standard deviation)

### Text processing

The collected clinical notes were written using Korean syntax. For topic modeling, words in the functional category were excluded. Therefore, we employed a Korean POS tagging program to sort meaningful tokens. The POS tagger used was the Korean Intelligent Word Identifier, developed through the 21st century Sejong Project [13].

Another issue was that the collected clinical notes contained many English terms. English terms representing diseases, symptoms, laboratory tests, etc. were used as tokens in their normalized forms. Finally, the same drug was referred to under different names. Drugs are represented by either their product names or their ingredient names in clinical notes. For example, “amlodipine,” which is named after its ingredient name, can be also called “Norvasc,” its product name. We replaced the product names with ingredient names to unify the different terms for the same drugs. Thus, the document frequency (DF) of drug names increased.

### Held-out test data

As stated above, 10% of each dataset was used as the test data. The remaining 90% of the dataset was used to train the classification model. This is contrary to the general convention of machinelearning projects that utilize dimensionality reduction. Conventionally, the test data are obtained after dimensionality reduction. However, in our study, the test data were held out before topic modeling to ensure that the classification model learned only the pattern inherent in the training data. This is essential because the model must forecast the onset of any future complications considering only the presence of clinical notes of patients with DM and the learned pattern in the training data. Table 2 presents the properties of the test data.

**Table 2:** Properties of Isolated Test Data

	M^*a*^	V^*b*^
DMR	341	5,762
DMN	197	4,569
NAFLD	85	2,431
CVA	81	2,719

^*a*^ number of merged documents ^*b*^ vocabulary size

### Topic modeling

For topic modeling, we used LDA-C, provided by David M. Blei [14] and translated it into Microsoft Visual. C#.NET 2022. The topic count was set to 100 because our unpublished preliminary study estimated that 100 was the optimal number of topics. First, a document-term matrix was created from the training data. Thereafter, it was converted into a document-topic matrix and topic-term matrix through topic modeling. The topic models can be optimized using two methods: Gibbs sampling and the EM algorithm. In this study, the EM algorithm was applied.

Next, the topic structure of the test data was estimated, considering the extracted topic structure of the training data. This process is called transplantation. Transplanting the topic models of the training data into the test data was necessary to match the dimensions of the topic structures of the two datasets. Matching the dimensions of the two structures is essential because the topic structure of the test data is inputted into the classification model. The model can compute the probabilities given a learned pattern in the training data when the input value has the same dimensions as the learned topic structure of the training data.

In the original LDA model proposed by Blei et al. [4], *γ* is a matrix (M × K) that represents the relationship between documents and topics. *ϕ* is a matrix (K × V) showing the relation between topics and words. *γ* is the feature set for a supervised machine learning project. The main concern of the transplantation in this study is, how to infer *γ* of the documents in the test data.

Therefore, we first check whether the nth word in the mth document in the test data, w_m,n_, is included in the test data in *ϕ which was estimated from train data.*. When w_m,n_ is the t-th word in *ϕ*, the weight value showing the relationship between the mth document and kth topic(*γ*_m,k_) can be calculated as follows:

$${\gamma }_{m, k}=\sum _{n}^{N}{TF}_{n}\times {e}^{{\varnothing }_{t,k}}$$

Here, N is the number of words in the mth document of the test data, and TFn is the term frequency of the nth word in the mth document. The second term expresses the rational number converted from the weight value of the tth word and kth topic in *ϕ* of the training data. Thereafter, we utilized the inferred *γ* as the feature set for supervised machine learning.

An important issue at this stage is the number of words appearing in the held-out test data that are absent from the transplanted topic model. These words are referred to as unseen data. Unseen data are those that the model has not yet learned. Therefore, they must be smoothed to improve the model quality. Consequently, the log-value of the unseen data was initialized to -100.0 to minimize its influence on calculating *γ* of the test dataset. Table 3 presents the percentage of unseen words included in the transplanted topic model for each held-out test dataset.

**Table 3:** Percentages of the words included in the transplanted topic model

Data	inTR(%)^*a*^	outTR(%)^*b*^
DMR	88.34	11.66
DMN	88.12	11.88
NAFLD	86.18	13.82
CVA	84.08	15.92

^*a*^ words which are included in the transplanted topic model,

^*b*^ words which are not included in the transplanted topic model

### Prediction models

Three prediction methods were used in this study: Random Forest (RF), Gradient Boosting Machine (GBM), and Extreme Gradient Boosting (XGBoost or XG). We utilized various R machine learning packages for the classification. We utilized “randomForest” package for R 4.2.1 for RF [15], “gbm” package for R 4.2.1 for GBM [16], and “xgboost” package for R 4.2.1 for XGBoost [17]. First, we performed a preliminary study of 10-fold cross-validation of each group of data. The ”caret” package for R 4.0.2 was utilized [18]. This preliminary study ensured the reliability of the prediction performance of the model. In this preliminary study, topic modeling was conducted prior to data segmentation. After topic modeling of the entire set, each group of data was divided into 10 parts. In each trial, using the nine parts as a training set, the remaining parts (i.e., the test set) were predicted. The test sets were rotated in a total of ten trials to ensure that every ten parts of the dataset were subject to prediction. As the main study, a held-out test was conducted for each group of data. As previously stated, the training set-test set ratio was set to 9:1. Contrary to the preliminary study, topic modeling was conducted after the training and test data were segmented.

## Results

In this section, we show the classification performance of both the preliminary and main studies. The accuracy metrics used in this section were precision, recall, F1 score, and specificity. Recall is mathematically equivalent to sensitivity. Therefore, it was not necessary to show the sensitivity separately.

Table 4 shows the average performance scores for predicting DMR, DMN, NAFLD, and CVA using a 10-fold cross-validation test. In the DMN group, the F1 scores for all three methods were greater than 0.9. In addition, the F1 score of DMR prediction ranged from 0.823 to 0.827 across the prediction methods. The NAFLD prediction was between 0.796 and 0.809. CVA prediction had the lowest range of F1 scores among the four disease types, which ranged between 0.742 and 0.781. The relatively low predictive performance of CVA and NAFLD may be attributable to the small size of the dataset. The F1 score of disease prediction for all three prediction methods was equal to or greater than 0.8, indicating the effectiveness of the model.

Table 5 presents the performance scores of the classifications for predicting DMR, DMN, NAFLD, and CVA using the held-out test. The F1 score for DMR prediction ranged from 0.84 to 0.86. DMN prediction was the highest among the four disease types, ranging between 0.918 and 0.925. The F1 scores for NAFLD prediction ranged from 0.805 to 0.831. Finally, the F1 score for CVA prediction ranged from 0.757 to 0.778. Similarly, the F1 score of disease prediction for all three prediction methods was near or greater than 0.8, indicating the effectiveness of the current approach.

**Table 4:** Averaged performance scores predicting DMR, DMN, NAFLD, and CVA before the isolation

Disease	Method	Precision	Recall	F1	Specificity
DMR	RF	0.897	0.763	0.824	0.907
	GBM	0.857	0.792	0.823	0.860
	XG	0.861	0.797	0.827	0.853
DMN	RF	0.937	0.895	0.915	0.942
	GBM	0.938	0.889	0.912	0.942
	XG	0.935	0.889	0.911	0.939
NAFLD	RF	0.858	0.770	0.809	0.883
	GBM	0.838	0.765	0.796	0.862
	XG	0.825	0.794	0.806	0.845
CVA	RF	0.832	0.673	0.742	0.865
	GBM	0.790	0.716	0.750	0.811
	XG	0.810	0.755	0.781	0.823

**Table 5:** Performance scores of classifications predicting DMR, DMN, NAFLD, and CVA from the topic models inferred from the held-out test data

Disease	Method	Precision	Recall	F1	Specificity
DMR	RF	0.897	0.794	0.842	0.904
	GBM	0.880	0.834	0.856	0.880
	XG	0.854	0.834	0.844	0.849
DMN	RF	0.977	0.866	0.918	0.980
	GBM	0.966	0.887	0.925	0.960
	XG	0.956	0.887	0.920	0.960
NAFLD	RF	0.868	0.750	0.805	0.878
	GBM	0.868	0.750	0.805	0.878
	XG	0.822	0.841	0.831	0.805
CVA	RF	0.875	0.700	0.778	0.902
	GBM	0.824	0.700	0.757	0.854
	XG	0.750	0.775	0.762	0.805

## Discussion

Our findings demonstrate that transplanting the topic model of the training data into the test data and entering the weighted topic structure of the test data as an input to the model can efficiently help predict the DM complications. As shown in Table 5, the performance scores for predicting complications of DM from the held-out test data were at an acceptable level. However, performance scores were non-uniform. Particularly, the performance scores for the prediction of CVA were lower than those for the other three complications.

Table 6 contains statistical data supporting the inference. In particular, it presents the inverse document frequency (IDF) values for all words in the four groups of training data. CVA had the smallest standard deviation of the IDF values among the four groups. It can be assumed that the small standard deviation of the IDF values of the CVA group implies poor quality of the topic model. However, we cannot conclusively determine whether this is the reason for the poor performance of the CVA prediction.

**Table 6:** Distributions of IDF values in all the training datasets

Data	DMR	DMN	NAFLD	CVA
**Skewness**	-1.450	-1.425	-1.583	-1.544
**Kurtosis**	1.831	1.756	2.394	2.252
**Min**	0.692	0.579	0.774	0.818
**1Qu** ^*a*^	5.829	5.282	4.855	4.797
**Median**	6.927	6.380	5.548	4.797
**Mean**	6.386	5.861	5.244	5.190
**3QU** ^*b*^	7.332	6.786	5.953	5.896
**Max**	7.324	6.786	5.953	5.896
**SD** ^*c*^	1.163	1.113	0.915	0.898

^*a*^ 1st quartile, ^*b*^ 3rd quartile, ^*c*^ standard deviation

Another possible answer arises from the scrutiny of the properties of held-out test data. Table 3 illustrates the percentages of unseen data. The percentage of unseen data was highest in the CVA data. A high percentage of unseen data constitutes poor conditions for machine learning with the inferred topic model. This may explain why CVA prediction was the poorest.

We cannot conclusively describe what determines the prediction performance. However, we suspect that the prediction performance is strongly related to data quality. Table 5 shows that the no-prediction method consistently outperforms the others. This implies that the quality of the data, rather than the prediction methods, is an important factor in determining prediction performance. Further studies on the factors affecting the prediction are required.

This approach also has certain advantages. Documents may not display textual overlap but still have an underlying thematic connection (i.e., overlap of topics). In our approach, the key information of our document collection was not extracted from the text but drawn through the estimation of its underlying topics. Therefore, flexible and data-driven analyses are possible. That is, the approach can detect a hidden pattern or structure underlying a set of data that is unknown to the field. During the initial stages of our study, we advised that NAFLD would be difficult to predict clinically. However, using the current approach, we could predict NAFLD with an F1 score > 0.8. Thus, our approach may identify unknown independent variables in a clinical prediction model.

However, this study has certain limitations. The first report of DM in the clinical notes collected from the SNUH EMR system did not coincide with the date of the first DM diagnosis. Specifically, some patients may have already been diagnosed with DM at a local clinic before visiting SNUH for the first time. Thus, in some marginal cases, the date a patient was diagnosed with a particular complication preceded the date they were diagnosed with DM at the SNUH. However, in the current study, these facts were disregarded, considering our currently available resources.

## Conclusion

In this study, by employing the training data, we trained the machine learning model to determine the pattern between topic structures and the future onset of complications. Thereafter, we transplanted the topic structure of the training data into that of the test data to match their dimensions. Finally, by inputting the resultant topic structure of the test data into the model, we developed a model to adequately predict the prognosis of DM complications. The prediction performance of DMN was the highest among the four groups of complications. However, the prediction performance of CVA was relatively low. This study showed that, by transplanting the topic models of the training data into the test data, it is possible to efficiently predict the probabilities of future complications from clinical notes.

### Electronic supplementary material

Below is the link to the electronic supplementary material.

Supplementary Material 1

## References

[1] 2018 WHO, Diabetes. 2018. https://www.who.int/news-room/fact-sheets/detail/diabetes. Accessed November 26 2022.

[2] Thomas PB, Robertson DH, Chawla NV (2018). Predicting onset of complications from diabetes: a graph based approach. Appl Netw Sci.

[3] Ljubic B, Hai AA, Stanojevic M, Diaz W, Polimac D, Pavlovski M, Obradovic Z (2020). Predicting complications of diabetes mellitus using advanced machine learning algorithms. J Am Med Inform Assoc.

[4] Blei DM, Ng AY, Jordan MI (2003). Latent dirichlet allocation. J Mach Learn Res.

[5] Blei DM (2012). Probabilistic topic models. Commun ACM.

[6] Papadimitriou CH, Raghavan P, Tamaki H, Vempala S (2000). Latent semantic indexing: a probabilistic analysis. J Comput Syst Sci.

[7] Hofman T. Probabilistic latent semantic indexing. ACM. 1999;50–7. 10.1145/312624.312649.

[8] Griffiths TL, Steyvers M (2004). Finding scientific topics. Proc Natl Acad Sci.

[9] Perotte A, Ranganath R, Hirsch JS, Blei D, Elhadad N (2015). Risk prediction for chronic kidney disease progression using heterogeneous electronic health record data and time series analysis. J Am Med Inform Assoc.

[10] Sarioglu E, Yadav K, Choi HA. Topic Modeling Based Classification of Clinical Reports, in: 51st Annual Meeting of the Association for Computational Linguistics Proceedings of the Student Research Workshop, pages, Sofia, Bulgaria, Association for Computational Linguistics, 2013:67–73. PMC10544137 37786783

[11] Restificar A, Ananiadou S. Inferring appropriate eligibility criteria in clinical trial protocols without labeled data. in: Proceedings of the ACM sixth international workshop on Data and text mining in biomedical informatics, 2012:21–28.

[12] Halpern Y, Horng S, Nathanson LA, Shapiro NI, Sontag D. A comparison of dimensionality reduction techniques for unstructured clinical text, in: ICML 2012 Workshop on Clinical Data Analysis, 2012.

[13] Korea:VANK TVANo 21c Sejong Project. http://sejong.prkorea.com/kor/main.jsp. 2022. Accessed December 26 2022.

[14] Blei D. in: LDA-c. 2016. https://github.com/blei-lab/lda-c. Accessed December 10 2022.

[15] Liaw A, Wiener M. Classification and regression by randomForest, R News 2002. 2002. https://cogns.northwestern.edu/cbmg/LiawAndWiener2002.pdf. Accessed December 26 2022.

[16] Greenwell B, Boehmke B, Cunningham J, Developers GGBM. Generalized Boosted Regression Models. 2018. https://cran.r-project.org/web/packages/gbm/index.html. Accessed December 26 2022.

[17] Chen T, He T, Benesty M, Khotilovich V, Tang Y, Cho H et al. Xgboost:extreme gradient boosting, R package version (4 – 2), 2015. https://cran.r-project.org/web/packages/xgboost/index.html. Accessed December 26 2022.

[18] Kuhn M (2008). Building predictive models in R using the caret package. J Stat Softw.

