# Supplementary material for: Prediction of complications in diabetes mellitus using machine learning models with transplanted topic model features
Source: Biomed Eng Lett. 2023 Oct 6;14(1):163–71. doi: 10.1007/s13534-023-00322-7 (PMC10769946; doi:10.1007/s13534-023-00322-7)
Supplement: Supplementary file 1 — Supplementary Material 1 [file 13534_2023_322_MOESM1_ESM.docx]

**Supplementary Table 1**. M × V matrix composed of 1,170 rows and 11,513 columns. This matrix is known as the term frequency matrix. M (1,170) is number of documents, V (11,513) means the number of words in the vocabulary. This matrix was used as input feature for Topic Modeling. Cells are filled with integer numbers.

|  | **Vocabulary (size: 11,513)** | | | | | | | |
| --- | --- | --- | --- | --- | --- | --- | --- | --- |
|  | V_1_ | V_2_ | V_3_ |  | V_11,510_ | V_11,511_ | V_11,512_ | V_11,513_ |
| **Document  (size: 1,770)** | IME | Breast | cancer | … | biphenyl | dictation | shiver | injure |
| M_1_ | 1 | 0 | 0 |  | 0 | 0 | 0 | 0 |
| M_2_ | 4 | 0 | 0 |  | 0 | 0 | 1 | 1 |
| M_3_ | 0 | 3 | 0 |  | 0 | 0 | 0 | 0 |
| M_4_ | 0 | 0 | 0 |  | 0 | 7 | 1 | 0 |
| M_5_ | 0 | 0 | 0 |  | 0 | 0 | 0 | 1 |
| M_6_ | 3 | 0 | 0 |  | 0 | 0 | 0 | 0 |
| M_7_ | 0 | 0 | 0 |  | 0 | 0 | 0 | 0 |
| M_8_ | 0 | 0 | 0 |  | 0 | 4 | 0 | 0 |
| M_9_ | 0 | 0 | 0 |  | 0 | 0 | 1 | 0 |
| M_10_ | 4 | 0 | 0 |  | 0 | 3 | 0 | 1 |
| … |  |  |  | … |  |  |  |  |
| M_1,689_ | 0 | 0 | 0 |  | 0 | 0 | 0 | 0 |
| M_1,690_ | 0 | 0 | 0 |  | 0 | 0 | 0 | 0 |
| M_1,770_ | 0 | 0 | 7 |  | 0 | 0 | 1 | 0 |

**Supplementary Table 2**. M × K matrix composed of 1,170 rows and 100 columns. M (1,170) is Number of Documents, V means number of vocabs. M means number of documents, K (100) means number of Topics. Cells are filled with real numbers. This matrix was used as input feature for machine learning algorithm such as Random Forest, Gradient boosting Machine, XG-boost.

|  | **Topic (size: 100)** | | | | | | | | |
| --- | --- | --- | --- | --- | --- | --- | --- | --- | --- |
| **Document  (size: 1,770)** | K_1_ | K_2_ | K_3_ | … | K_7_ | K_8_ | K_9_ | K_100_ |  |
| M_1_ | 5.5175 | 2.8968 | 4.3124 |  | 2.3848 | 3.1510 | 2.5470 | 2.3046 |  |
| M_2_ | 1.4491 | 1.5281 | 1.3507 |  | 1.5915 | 1.5920 | 1.3712 | 1.1990 |  |
| M_3_ | 3.3102 | 2.5653 | 3.0228 |  | 3.1250 | 2.7201 | 2.3130 | 2.4743 |  |
| M_4_ | 6.0971 | 16.9182 | 3.9404 |  | 3.7440 | 5.1024 | 5.3580 | 4.5684 |  |
| M_5_ | 6.5005 | 10.0689 | 7.7715 |  | 5.8074 | 8.4794 | 6.7063 | 6.7003 |  |
| M_6_ | 11.3213 | 9.4850 | 9.0234 |  | 8.2764 | 9.0387 | 8.3746 | 9.4336 |  |
| M_7_ | 2.1908 | 1.9359 | 1.8466 |  | 2.1137 | 2.3409 | 2.3830 | 1.6764 |  |
| M_8_ | 1.0057 | 1.2402 | 1.1684 |  | 1.2595 | 1.7681 | 1.2277 | 1.0199 |  |
| M_9_ | 3.3690 | 4.5910 | 3.5190 |  | 2.8016 | 4.0630 | 3.5383 | 4.4836 |  |
| M_10_ | 7.2494 | 9.2728 | 5.4781 |  | 5.5803 | 5.7869 | 6.4034 | 5.6033 |  |
| … |  |  |  | … |  |  |  |  |  |
| M_1,689_ | 2.7249 | 2.3138 | 2.2401 |  | 2.5084 | 2.7194 | 2.7564 | 2.1730 |  |
| M_1,690_ | 5.2703 | 5.2852 | 5.1666 |  | 4.0540 | 5.3204 | 4.5617 | 5.1242 |  |
| M_1,770_ | 19.6600 | 26.8956 | 14.8155 |  | 12.8373 | 15.6945 | 18.5603 | 32.3179 |  |
